# Editorial: Leveraging physical activity for disease prevention and management in special populations

**DOI:** 10.3389/fspor.2026.1925441

**Published:** 2026-08-13

**Authors:** Georgian Badicu, Felipe J. Aidar

**Affiliations:** 1 Department of Physical Education and Special Motricity, Transilvania University of Brasov, Brasov, Romania; 2 Department of Physical Education, Federal University of Sergipe (UFS), São Cristóvão, Brazil

**Keywords:** disease, exercise, physical activity, prevention, special populations

Physical activity (PA), defined as any bodily movement produced by skeletal muscles that results in energy expenditure, is widely recognized as a cornerstone of health promotion and disease prevention. Exercise, in contrast, represents a structured, planned, and repetitive subtype of physical activity specifically designed to improve or maintain physical fitness and health (1). Both regular physical activity and appropriately prescribed exercise contribute substantially to improving cardiovascular, metabolic, musculoskeletal, neurological, and psychological health across the lifespan (2–4). Nevertheless, physical inactivity remains a major global public health challenge. According to the World Health Organization, nearly one-third of adults worldwide do not meet the recommended levels of physical activity, contributing substantially to the global burden of non-communicable diseases, premature mortality, and escalating healthcare costs (4, 5). The most recent global pooled analysis, based on 507 population-based surveys including 5.7 million participants from 2000 to 2022, confirms that approximately 31% of adults worldwide do not meet the World Health Organization (WHO) physical activity recommendations, with persistent regional and sex-related disparities highlighting the ongoing need for effective public health interventions (6). These observations highlight the urgent need for effective strategies that promote both habitual physical activity and evidence-based exercise interventions tailored to diverse populations.

Special populations—including individuals living with chronic diseases, neurological disorders, functional impairments, obesity, age-related conditions, or other health limitations—present unique physiological, functional, and clinical characteristics that often require individualized exercise prescription rather than generic recommendations. Consequently, the concept of *Exercise is Medicine* has evolved toward precision exercise medicine, emphasizing personalized exercise programs based on scientific evidence, clinical characteristics, functional capacity, and individual goals (7, 8). In parallel, advances in digital health technologies, wearable sensors, rehabilitation sciences, and implementation research have created new opportunities to objectively monitor physical activity, optimize exercise prescription, evaluate intervention effectiveness, and facilitate long-term adherence. Together, these developments support a more personalized, evidence-informed approach to disease prevention, rehabilitation, and long-term health management in special populations.

The present Research Topic, *Leveraging Physical Activity for Disease Prevention and Management in Special Populations*, was established to showcase contemporary evidence on the role of physical activity and structured exercise interventions in disease prevention, rehabilitation, functional recovery, and long-term health management across diverse clinical populations (4, 5). Although the six published articles address distinct populations and research questions, they contribute to the expanding body of literature demonstrating the transition from generalized physical activity recommendations toward personalized, evidence-based exercise prescription, while providing additional evidence across diverse clinical populations. Together, they reinforce the concept that exercise should not be regarded merely as an adjunct to conventional healthcare but rather as a core component of prevention, rehabilitation, and chronic disease management, adapted to the physiological, functional, and clinical characteristics of each population. The Research Topic encompasses a broad spectrum of exercise science, ranging from systematic evidence on the effects of physical activity on depressive symptoms in middle-aged and younger-old adults (Xia et al.) to rehabilitation strategies for peripheral neuropathy (Zhao et al.), mitochondrial myopathy (Wan et al.), Parkinson's disease (Smith and Hellman), and asthma (Song et al.), as well as mechanistic insights into exercise-induced myokines and their antitumor potential (Han et al.). Collectively, these contributions illustrate the multidisciplinary nature of exercise medicine by integrating clinical rehabilitation, neuroscience, oncology, respiratory medicine, mental health, and molecular biology. More importantly, they demonstrate how complementary research approaches—including randomized interventions, systematic reviews, meta-analyses, and mechanistic syntheses—can collectively strengthen the evidence base for translating exercise science into personalized clinical practice and public health strategies.

The systematic review and meta-analysis by Xia et al. provides important evidence regarding the relationship between physical activity and mental health in middle-aged and younger-old adults experiencing depressive symptoms. By synthesizing randomized controlled trials, the authors demonstrate that structured exercise interventions, particularly long-term Tai Chi programs performed at least three times per week for more than six months, were associated with greater reductions in depressive symptoms among medication-free participants. Beyond reporting intervention effectiveness, the study highlights the importance of behavioral and psychological factors—including adherence, motivation, and sustained participation—in determining long-term intervention success. These findings reinforce the growing recognition that effective exercise prescription should integrate both physiological and psychosocial dimensions to maximize health outcomes.

Complementing these findings, Zhao et al. demonstrate the therapeutic potential of targeted exercise interventions for older adults with peripheral neuropathy. Their randomized trial showed that an eight-week Tai Chi program produced greater improvements in plantar tactile sensation and ankle proprioception than brisk walking, suggesting that exercise modality plays a critical role in optimizing sensorimotor rehabilitation. These results emphasize that exercise prescription should extend beyond general recommendations for physical activity by considering disease-specific impairments and selecting interventions capable of addressing underlying functional deficits. Such evidence supports the growing movement toward personalized rehabilitation strategies based on the clinical characteristics of individual patients.

At the mechanistic level, the review by Han et al. expands current understanding of how exercise exerts protective effects through molecular pathways. By synthesizing evidence on exercise-induced myokines—including IL-6, SPARC, irisin, and other muscle-derived signaling molecules—the authors illustrate how skeletal muscle functions as an endocrine organ capable of modulating inflammation, immune responses, and tumor progression. Importantly, the review also identifies significant knowledge gaps, including the limited availability of clinical validation studies and the need to better characterize intracellular signaling pathways underlying these biological effects. This mechanistic perspective strengthens the scientific rationale for integrating exercise into precision medicine while highlighting priorities for future translational research.

The systematic review by Wan et al. further strengthens the evidence supporting exercise as a therapeutic strategy for individuals with rare neuromuscular disorders by evaluating exercise training in patients with mitochondrial myopathy. The review demonstrates that moderate-intensity aerobic and resistance exercise, particularly when combined, can improve aerobic capacity, muscle strength, mitochondrial enzyme activity, and oxidative metabolism without consistently increasing markers of muscle damage. Importantly, the authors emphasize that exercise responses vary according to genetic phenotype, highlighting the necessity of phenotype-specific exercise prescription r**a**ther than uniform rehabilitation protocols. This work reinforces the growing paradigm of precision exercise medicine, in which exercise interventions are tailored to the biological and clinical characteristics of individual patients.

Similarly, the systematic review by Smith and Hellman expands the scope of exercise rehabilitation by examining climbing-based interventions for individuals with Parkinson's disease. Although the available evidence remains limited, the review suggests that therapeutic climbing is a feasible and acceptable intervention capable of improving motor function while simultaneously engaging multiple components of physical fitness, including strength, balance, flexibility, and neuromotor control. The authors also identify important gaps in the current literature, particularly the need for larger randomized controlled trials, standardized intervention protocols, and greater attention to patient-reported outcomes and non-motor symptoms. Their findings illustrate how innovative exercise modalities may complement conventional rehabilitation strategies while broadening opportunities for individualized patient care.

From a broader evidence-based perspective, Song et al. synthesize the comparative effectiveness of different exercise modalities for improving asthma-related quality of life through a systematic review and network meta-analysis. Their findings indicate that comprehensive exercise programs combining aerobic exercise with breathing or resistance training consistently outperform single-modality interventions, highlighting the value of multimodal rehabilitation strategies for chronic respiratory disease. Beyond identifying the most effective intervention approaches, the study demonstrates how advanced evidence synthesis can support clinical decision-making, optimize exercise prescription, and inform future guideline development by integrating both direct and indirect comparative evidence. Collectively, these findings reinforce the importance of tailoring exercise interventions not only to specific diseases but also to the functional and clinical needs of individual patients.

Although the six articles included in this Research Topic investigate distinct clinical conditions and employ diverse methodological approaches, several overarching themes emerge that collectively advance the field of exercise medicine. First, they consistently demonstrate that exercise interventions, when appropriately prescribed, can improve outcomes across a broad spectrum of special populations, including individuals with depression, peripheral neuropathy, mitochondrial myopathy, Parkinson's disease, asthma, and cancer-related conditions. Second, the findings emphasize that the effectiveness of exercise depends not only on its physiological effects but also on the careful alignment of intervention characteristics—including modality, intensity, frequency, duration, and progression—with the functional capacity, disease status, and individual goals of each patient (2, 4, 8). This growing body of evidence reinforces the paradigm shift from generalized physical activity recommendations toward personalized and evidence-based exercise prescription (7, 8).

Another important message emerging from this Research Topic is the increasingly multidisciplinary nature of exercise medicine. The included studies collectively demonstrate that optimizing health outcomes requires collaboration among clinicians, rehabilitation specialists, physiotherapists, psychologists, exercise physiologists, public health researchers, and implementation scientists. At the same time, they highlight the need to bridge the gap between efficacy demonstrated under controlled experimental conditions and effectiveness achieved in routine clinical practice. Individuals with chronic diseases or disabilities frequently encounter barriers to participation in exercise programs, including functional limitations, multimorbidity, reduced access to supervised rehabilitation, socioeconomic constraints, and adherence challenges (2, 4, 8, 9).Consequently, future efforts should focus not only on developing effective interventions but also on designing feasible, scalable, and accessible programs capable of being successfully implemented across diverse healthcare and community settings.

Looking forward, several priorities for future research emerge from this Research Topic. Advances in wearable technologies, digital health platforms, artificial intelligence, and remote monitoring systems offer unprecedented opportunities to personalize exercise prescription, objectively monitor adherence, and evaluate intervention effectiveness in real-world settings. However, further longitudinal and implementation studies are needed to clarify dose–response relationships, optimize long-term adherence, evaluate cost-effectiveness, and determine how exercise interventions can be successfully integrated into routine healthcare. Particular attention should also be devoted to underrepresented populations, health equity, and the development of precision exercise medicine approaches that remain accessible across different healthcare systems and socioeconomic contexts.

In conclusion, the studies gathered in this Research Topic collectively demonstrate that exercise is no longer simply a complementary lifestyle strategy but an evidence-based therapeutic intervention capable of preventing disease, supporting rehabilitation, improving functional capacity, and enhancing quality of life across diverse special populations**.** By integrating mechanistic research, randomized clinical interventions, systematic reviews, and meta-analyses, this collection provides a comprehensive perspective on the expanding role of exercise in contemporary healthcare. We hope these contributions will stimulate further interdisciplinary collaboration, encourage the development of personalized exercise interventions, and support the continued integration of exercise medicine into clinical practice, rehabilitation, and public health policies worldwide.
